# Prediction of Biochemical Recurrence-Free Survival of Prostate Cancer Patients Leveraging Multiple Gene Expression Profiles in Tumor Microenvironment

**DOI:** 10.3389/fonc.2021.632571

**Published:** 2021-09-23

**Authors:** Rui Zhou, Yuanfa Feng, Jianheng Ye, Zhaodong Han, Yuxiang Liang, Qingbiao Chen, Xiaoming Xu, Yuhan Huang, Zhenyu Jia, Weide Zhong

**Affiliations:** ^1^ School of Medicine, South China University of Technology, Guangzhou, China; ^2^ Department of Urology, Guangdong Key Laboratory of Clinical Molecular Medicine and Diagnostics, Guangzhou First People’s Hospital, School of Medicine, South China University of Technology, Guangzhou, China; ^3^ Affiliated Foshan Hospital of Southern Medical University, Southern Medical University, Foshan, China; ^4^ Department of Urology, Hwa Mei Hospital, University of Chinese Academy of Sciences, Ningbo, China; ^5^ Department of Microbiology, Immunology, and Molecular Genetics, University of California, Los Angeles, Los Angeles, CA, United States; ^6^ Department of Botany and Plant Sciences, University of California, Riverside, Riverside, CA, United States

**Keywords:** prostate cancer, microenvironment, tumor-adjacent normal tissue, recurrence-free survival, prognosis

## Abstract

Tumor-adjacent normal (TAN) tissues, which constitute tumor microenvironment and are different from healthy tissues, provide critical information at molecular levels that can be used to differentiate aggressive tumors from indolent tumors. In this study, we analyzed 52 TAN samples from the Cancer Genome Atlas (TCGA) prostate cancer patients and developed a 10-gene prognostic model that can accurately predict biochemical recurrence-free survival based on the profiles of these genes in TAN tissues. The predictive ability was validated using TAN samples from an independent cohort. These 10 prognostic genes in tumor microenvironment are different from the prognostic genes detected in tumor tissues, indicating distinct progression-related mechanisms in two tissue types. Bioinformatics analysis showed that the prognostic genes in tumor microenvironment were significantly enriched by p53 signaling pathway, which may represent the crosstalk tunnels between tumor and its microenvironment and pathways involving cell-to-cell contact and paracrine/endocrine signaling. The insight acquired by this study has advanced our knowledge of the potential role of tumor microenvironment in prostate cancer progression.

## Introduction

Prostate cancer, which is one of the most common and deadly tumors in men, represents over 20% of newly diagnosed male cancers every year (1). In 2019, there were about 191,930 diagnosed cases and 33,330 deaths of prostate cancer in the USA (1). Although prostate cancer has the best 5-year survival rate among all types of cancers, about one-fourth of diagnosed patients are subject to high risk of postsurgery recurrence that threatens lives, making prostate cancer the second leading cause of cancer death in men in the USA (2, 3).

Disease screening, including prostate-specific antigen (PSA) test and digital rectal exam (DRE), and pathological characterization of biopsy tissues substantially contribute to the diagnosis and early risk stratification for patients with prostate cancer. For the patients treated with prostatectomy, a prognosis is needed to assess the risk of biochemical recurrence (BCR) for the development of further therapy plans. Various nomograms have been devised to predict postsurgery BCR-free survival primarily based on pathological variables, including Gleason score, tumor grade, and tumor stage. However, these traditional nomogram models had limited prediction accuracy due to large variation in scoring of these pathological variables as well as the heterogeneous nature of prostate cancer. With the rapid advancement of the technologies for quantification of genomic data, numerous biomarkers have been developed for predicting the outcomes of prostate cancer, for example, urine PCA3 (4), transmembrane protease, serine 2-TMPRSS2-ERG fusion (5), and a few commercial tests based on the profiles of multiple genes, including prolaris and decipher (6, 7). These new prognostic schemes using biomarkers, which have gain ground in the clinical application (8), are based on the assay of tumor tissue samples; thus, their prediction accuracies are potentially still hampered by tumor heterogeneity of various levels.

Tumor microenvironment is not an inert component; rather, known as a battlefield between the cancer cells and stromal cells, it plays an important role in cancer progression and metastasis (9). Tumor-adjacent tissues actively interact with tumor through the extracellular matrix or secreted factors, to either fight against tumor or to facilitate tumor growth when these tissues have been domesticated by tumor. Although tumor-adjacent tissues appear histologically normal, alterations in genomic transcription have been identified between tumor-adjacent tissues and authentic normal prostate tissues from disease-free subjects, which have been applied as a diagnostic tool to detect the presence of tumor even if biopsy samples do not contain tumor (10). A set of 15 genes have been characterized to be specifically activated in histologically normal tissue adjacent to various types of tumor based on the analysis of the Cancer Genome Atlas (TCGA) database (11). It has been also shown that expression profiles of certain genes in tumor-adjacent tissue may reflect the characteristics of the tumor, either aggressive or indolent; such genes may be useful to the prediction of tumor outcomes including BCR (12). A recent study on breast cancer also suggested that tumor microenvironment provided useful information in understanding disease recurrence, which can be used to guide the development of surgical strategies (13). Therefore, tumor-adjacent tissues are as important as tumor tissues in cancer research to advance our knowledge of cancer biology. Due to their more homogeneous genetic background, tumor-adjacent tissues may serve as better clinical material for disease prognosis than tumor tissues. A literature search indicated that majority of the prognosis studies in prostate cancer were focused on tumor tissues, while TAN tissues have been understudied. Moreover, lacking authentic normal prostate tissues in most of these prostate cancer studies, TAN tissues were often improperly employed as control to address the differences between diseased tissues and healthy tissues (14, 15).

In this study, the dataset from the Cancer Genome Atlas Prostate Adenocarcinoma (TCGA-PRAD) project and the dataset E-MTAB-6128, which represent the only data sources with TAN samples, were used to develop and validate a prognostic model based on expression profiles of multigene in tumor microenvironment. Only TAN prostate tissues from these two datasets have been analyzed in this study to establish a multigene model that can accurately predict the BCR-free survival for patients. First, the differentially expressed genes (DEGs) were identified between patients who experienced BCR and the BCR-free patients in the training set, i.e., TCGA-PRAD project. Gene Ontology (GO), Kyoto Encyclopedia of Genes and Genomes (KEGG) pathway analysis, and Protein-Protein Interaction (PPI) Network analysis were utilized to explore the potential connections among these BCR-related DEGs in TAN tissues. The univariate Cox regression analysis and the least absolute shrinkage and selection operator (LASSO) analysis were performed to further screen these DEGs, yielding a 10-gene prognostic signature based on TAN tissues. The potential functions or relatedness among these 10 signature genes were further demonstrated by the gene-set enrichment analysis (GSEA). Finally, the prediction accuracy of this new 10-gene prognostic model, specifically devised for the tests based on TAN tissues, has been validated using TAN samples from an independent patient cohort, i.e., E-MTAB-6128.

## Method

### Prostate Cancer Data and Preprocessing

High-throughput RNA-sequencing count data and clinical data of prostate cancer patients were downloaded from TCGA-PRAD project using GDCRNAtools (16). From a total of 547 TCGA patient samples, only 52 TAN samples were selected for this study. An independent dataset (E-MTAB-6128), which consisted of mRNA counts data and clinical data of prostate cancer patients were obtained from the ArrayExpress (https://www.ebi.ac.uk/arrayexpress/) (17). From a total of 141 E-MTAB-6128 patient samples, only 26 TAN samples were used for validation. The potential batch effect (unwanted systematic bias) between two datasets was eliminated using the function of “removeBatchEffect” in “limma” package in R. Trimmed mean of *M* value (TMM) normalization of the count data was performed using edgeR. The clinical characteristics for the prostate cancer patients in both datasets are summarized in **Table 1**.

**Table 1:** Clinical characteristics of selected sample in TCGA-PRAD project, E-MTAB-6128, and pooled cohort.

Factor	TCGA-PRAD cohort	E-MTAB-6128 cohort	Pooled cohort
Number	52	26	78
Age (mean ± SD)	60.28 ± 7.32	62.73 ± 5.69	61.1 ± 6.88
Gleason score			
≤6	6	19	25
7	40	4	44
≥8	17	3	20
AJCC pathologic T stage			
T2	29	23	52
T3	21	3	24
T4	2	–	
Preoperative PSA (ng/ml)			0
≤4	6	4	10
>4	46	21	67
Recurrence-free survival			
Yes	5	4	9
No	47	22	69

### Differential Expressed Gene Analysis

The DEGs in TAN tissues between patients who experienced BCR and the BCR-free patients were identified using edgeR package. A twofold change or more and *p* < 0.05 were used as the selection criterions for the identification of DEGs.

### Biological Enrichment and PPI Network Analysis

The GO assay and KEGG pathway analysis were employed to analyze the BCR-related DEGs through clusterProfiler package (18). The *p*-value and adjusted *p*-value by the Benjamin-Hochberg method were calculated for each identified pathway. The PPI interaction network among these BCR-related DEGs was established by STRING and visualized by Cytoscape (19).

### Selection of Prognostic Genes and Construction of Prognostic Model

The univariate Cox regression analysis was first employed to test the association between BCR-free survival and the profile of each identified DEG. The LASSO method was implemented by the glmnet package in R to screen the survival-relevant genes, with BCR status (1 for BCR and 0 for BCR-free) being treated as binary response variable. The optimal lambda value in the LASSO model was determined by cross-validation to achieve a minimum estimation error. Finally, the genes with nonzero coefficients were selected by LASSO analysis for the construction of a prognostic model based on their expression in TAN tissues. The linear combination formula for calculating a risk score (RS) of a patient using this prognostic model is


$$\text{RS}=\sum_{i=1}^{n}X_{i}\beta_{i},$$

where RS is the risk score of BCR for a patient, *n* is the number of genes included in this prognostic model, *X_i_* is the expression level of the *i*th gene measured in the patient’s TAN sample, and *β_i_* represents the regression coefficient of the *i*th gene estimated by LASSO method. The Kaplan-Meier analysis and log-rank test were used to test the association between the BCR-free survival and the RSs calculated by the TAN-tissue-based prognostic model, with patients being dichotomized into high-risk and low-risk groups with the median value of the RSs. The *p* < 0.05 was adopted as the significance level for these tests. The receiver operating characteristic (ROC) curve and area under the curve (AUC) were used to evaluate the prediction accuracy of the model using “survivalROC” package.

### Validation of the Prognostic Model With an Independent Cohort

Using the exact prognostic model trained by the TCGA-PRAD, we calculated the RS for the patients in the validation set (E-MTAB-6128) based on the TAN samples. These validation patients were also dichotomized into high- and low-risk groups based on the median RS value. The Kaplan-Meier analysis was used to test the association between BCR-free survival and the RS. The ROC curve and AUC were used to assess the prediction accuracy of the prognostic model.

### Biological Function Analysis of the Genes in the Prognostic Model

To further explore the potential functions of the prognostic genes in the model, we divided the TAN samples of TCGA-PRAD dataset into high- and low-risk groups based on the median RS values calculated by the model, and then performed the differentially expressed analysis between these two groups using edgeR package. All the genes (transcriptome) were sorted by the log2 (fold change) in decreasing order, and then analyzed by the GSEA analysis in R package clusterProfilter using “hallmark gene sets”, “KEGG gene sets”, and “Ontology gene sets” as the annotation sets. These annotation gene sets were downloaded from the Molecular Signatures Database (MSigDB).

## Result

### Identification of the Differentially Expressed Genes Between Patients Who Experienced BCR and BCR-Free Patients Using TAN Samples

**Figure 1:**
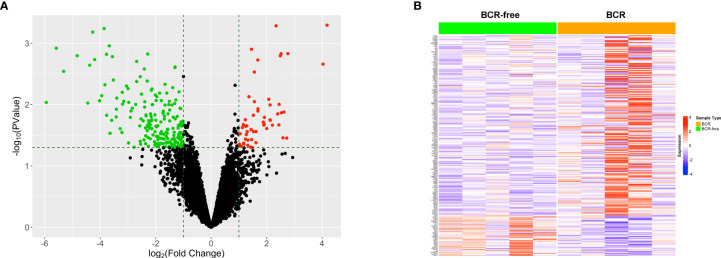
Identification of the differentially expressed genes between patients who experienced BCR and BCR-free patients using tumor-adjacent normal samples. **(A)** Volcano plot for the differentially expressed genes. The red/green dot represents upregulated/downregulated genes in the BCR group. The horizontal dashed line represents the cutoff of *p* < 0.05. The two vertical dashed lines denote the cutoffs of log2 FC <−1 (left) or log2 FC >1 (right), respectively. **(B)** Heatmap showing the profiles of the differentially expressed genes in TAN tissues between BCR patients and BCR-free patients.

The data for 52 prostate TAN tissue samples, including RNAseq data and patients’ clinical data, were selected from TCGA-PRAD project and used as the training set. Five out of these 52 patients experienced BCR after the surgery (**Table 1**), forming the BCR group. From the remaining 47 patients, we selected another five patients who had the greatest BCR-free survival times to constitute the BCR-free group. The differential expression analysis was used to identify 223 DEGs between these two groups based on the selection criteria of fold change >2 and *p* < 0.05 (**Figure 1A**). The heatmap in **Figure 1B** shows the expression profiles of these DEGs between BCR patients and BCR-free patients.

### Enrichment Analysis of the Differentially Expressed Genes

**Figure 2:**
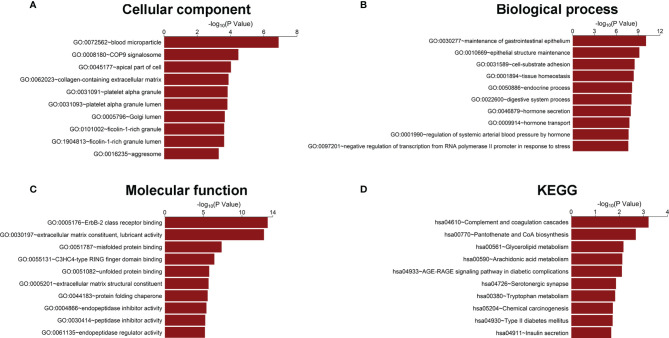
Gene Ontology and KEGG enrichment analysis of DEGs. **(A)** Cellular component, **(B)** biological process, **(C)** molecular function, and **(D)** KEGG pathway analysis.

To further explore the potential connection between the genes in our model and prostate cancer progression, all the 223 DEGs identified between the BCR group and the BCR-free group in the training set were analyzed using the GO and KEGG methodologies to mine the potential pathways or associated biological characteristics represented by these genes. The results from the GO analysis showed that the DEGs were enriched in “COP9 signalosome” and “collagen-containing extracellular matrix” in cellular component (**Figure 2A**); “cell-substrate adhesion,” “hormone secretion”, and “hormone transport” in biological process (**Figure 2B**); “ErbB-2 class receptor binding” and “extracellular matrix constituent, lubricant activity” in molecular function (**Figure 2C**), and “pantothenate and CoA biosynthesis” and “tryptophan metabolism” in KEGG (**Figure 2D**).

### PPI Network Analysis of the Differentially Expressed Genes

**Figure 3:**
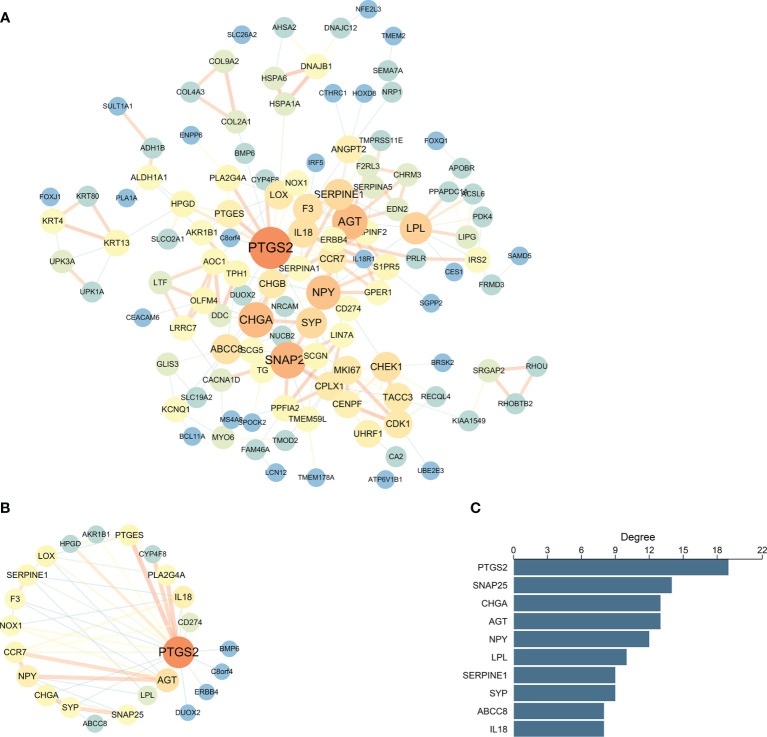
PPI network analysis of the DEGs. **(A)** The connection among DEGs. **(B)** Sub-PPI network of the top 10 hub genes in DEGs. The larger circles and blue to pink color correspond to the higher degrees between genes, and the wider line and blue to pink color refer to the increasing of the combined score. **(C)** The levels of degree for the top 10 hub genes in DEGs.

A total of 52 nodes and 448 interaction pairs were identified in the PPI network (**Figure 3A**). The nodes with high topological scores may play important roles in the disease. In this study, the top 10 nodes (degree ≥8), including prostaglandin-endoperoxide synthase 2 (PTGS2), synaptosome-associated protein 25 (SNAP25), chromogranin A (CHGA), angiotensinogen (AGT), neuropeptide Y (NPY), lipoprotein lipase (LPL), serpin family E member 1 (SERPINE1), synaptophysin (SYP), ATP-binding cassette subfamily C member 8 (ABCC8), and interleukin 18 (IL-18) were regarded to be the hub nodes of the network (**Figure 3C**). The subnetwork of these 10 hub nodes and their interaction pairs is shown in **Figure 3B**.

### Development of Prognostic Models Based on Gene Expression Profiles in Tumor Microenvironment

**Figure 4:**
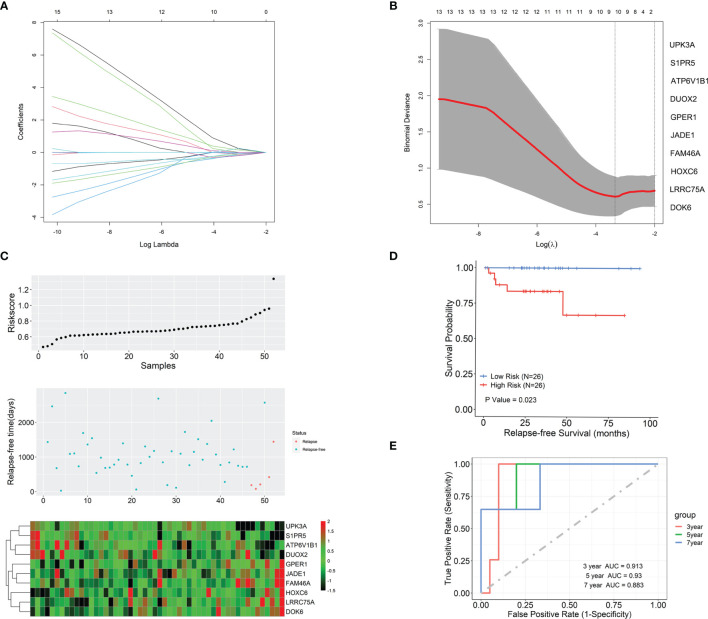
The development of the prognostic model based on gene expression profiles in tumor microenvironment (TAN) samples and the evaluation of this model. **(A)** LASSO coefficient profiles of the 10 prognostic TAN-related genes. **(B)** Selection of the optimal lambda in the LASSO model. **(C)** The risk score, survival status, and expression abundances of the 10 genes based on the TAN samples in TCGA-PRAD project. **(D)** Kaplan-Meier survival analysis in terms of BCR-free survival for the high- and low-risk patient groups. **(E)** The ROC analysis for the prediction of the 3-, 5-, and 7-year BCR-free survival based on the risk scores calculated by the 10-gene prognostic model.

The univariate Cox regression analysis was first used to analyze the association between each of the 223 DEGs and BCR-free survival based on the 52 TAN samples. A total of 25 out of these 223 genes in tumor microenvironment were detected to be significantly relevant to BCR-free survival using the threshold of *p* < 0.05 (**Table 2**). From these 25 potential prognostic genes, the analysis of the 52 TAN samples using LASSO identified 10 final genes to establish a prediction model, with the optimal lambda value being set to 0.036 (**Figures 4A, B**). These 10 genes included Jade family PHD finger 1 (JADE1), uroplakin 3A (UPK3A), family with sequence similarity 46 member A (FAM46A), ATPase H+ transporting V1 subunit B1 (ATP6V1B1), dual oxidase 2 (DUOX2), G protein-coupled estrogen receptor 1 (GPER1), sphingosine-1-phosphate receptor 5 (S1PR5), leucine-rich repeat containing 75A (LRRC75A), homeobox C6 (HOXC6), and docking protein 6 (DOK6). Wilcoxon signed-rank test was conducted on these 10 genes between TAN tissues and corresponding prostate cancer tissues in TCGA-PRAD cohort. As shown in **Supplementary Figure S1**, FAM46A, ATP6V1B1, DUOX2, GPER1, S1PR5, and HOXC6 were significantly differentially expressed between TAN tissues and corresponding prostate cancer tissues (Wilcoxon signed-rank test, *p* < 0.05), while JADE1, LRRC75A, UPK3A, and DOK6 did not show statistically differential expression between these two types of tissues (Wilcoxon signed-rank test, *p* > 0.05). The linear combination formula of the prognostic model for the calculation of the risk scores (RS) using the expression values of these 10 genes is


(1) $$\begin{array}{c} \text{RS}=X_{\text{JADE}1}\times 0.0048+X_{\text{UPK}3\text{A}}\times (-0.0144)+X_{\text{FAM}46\text{A}}\times 0.0326+X_{\text{ATP}6\text{V}1B1}\times (-0.0206)+\text{XDUOX}2\times (-0.0091)\\ +\;X_{\text{GPER}1}\times 0.0668+X_{\text{S}1\text{PR}5}\times (-0.0054)+X_{\text{LRRC}75A}\times 0.0020+X_{\text{HOXC}6}\times 0.0246+X_{\text{DOK}6}\times 0.0337 \end{array}$$

**Table 2:** Univariate Cox regression analysis of DEGs.

Gene	Symbol	Coef	HR	Lower 95	Upper 95	*p*-Value
ENSG00000109101	FOXN1	−1.003	0.367	0.169	0.797	0.011
ENSG00000181350	LRRC75A	1.239	3.452	1.317	9.050	0.012
ENSG00000204618	RNF39	−1.402	0.246	0.082	0.742	0.013
ENSG00000180739	S1PR5	−0.677	0.508	0.298	0.867	0.013
ENSG00000196754	S100A2	−0.511	0.600	0.400	0.901	0.014
ENSG00000206052	DOK6	0.681	1.976	1.141	3.420	0.015
ENSG00000121552	CSTA	−0.570	0.566	0.357	0.897	0.015
ENSG00000127129	EDN2	−1.064	0.345	0.142	0.840	0.019
ENSG00000100373	UPK3A	−0.386	0.680	0.492	0.940	0.019
ENSG00000171401	KRT13	−0.568	0.567	0.347	0.924	0.023
ENSG00000197757	HOXC6	0.462	1.588	1.062	2.374	0.024
ENSG00000136943	CTSV	−1.543	0.214	0.055	0.823	0.025
ENSG00000135414	GDF11	0.804	2.234	1.103	4.526	0.026
ENSG00000140279	DUOX2	−0.542	0.582	0.360	0.941	0.027
ENSG00000119866	BCL11A	−0.779	0.459	0.228	0.924	0.029
ENSG00000221818	EBF2	0.383	1.467	1.038	2.073	0.030
ENSG00000164850	GPER1	0.916	2.498	1.088	5.736	0.031
ENSG00000112773	FAM46A	0.669	1.953	1.045	3.648	0.036
ENSG00000160326	SLC2A6	1.265	3.541	1.063	11.796	0.039
ENSG00000124935	SCGB1D2	−0.684	0.505	0.260	0.980	0.043
ENSG00000077684	JADE1	1.014	2.756	1.027	7.399	0.044
ENSG00000108352	RAPGEFL1	−1.477	0.228	0.054	0.963	0.044
ENSG00000043039	BARX2	−0.704	0.495	0.249	0.984	0.045
ENSG00000116039	ATP6V1B1	−0.543	0.581	0.341	0.990	0.046
ENSG00000091879	ANGPT2	0.840	2.316	1.016	5.276	0.046

where RS is the BCR risk score for a patient and *X*_g_ represents the expression level of gene g measured in the patient’s TAN sample. With this formula, the risk scores for 52 patients in TCGA-PRAD cohort were calculated. The distribution of the risk scores, survival status, and expression abundances of 10 genes are shown in **Figure 4C**. It appeared that GPER1, JADE1, FAM46A, HOXC6, LRRC75A, and DOK6 were positively correlated with the risk score and recurrence status, while UPK3A, S1PR5, ATP6V1B1, and DUOX2 were negatively associated with risk score and recurrence status. The patients were then dichotomized into a high-risk group and a low-risk group, with equal size, by the median-risk score (0.672). In **Figure 4D**, Kaplan-Meier analysis showed that patients in the high-risk group had significantly worse clinical outcomes than those in the low-risk group (*p* = 0.023), in regard to BCR. The ROC analysis indicated that, based on these 52 patients in TCGA-PRAD project, the prognostic accuracies for 3-, 5-, and 7-year BCR-free survival were 0.913, 0.93, and 0.883, respectively, using this prognostic model (**Figure 4E**).

### Validation of the 10-Gene Prognostic Model for TAN samples

**Figure 5:**
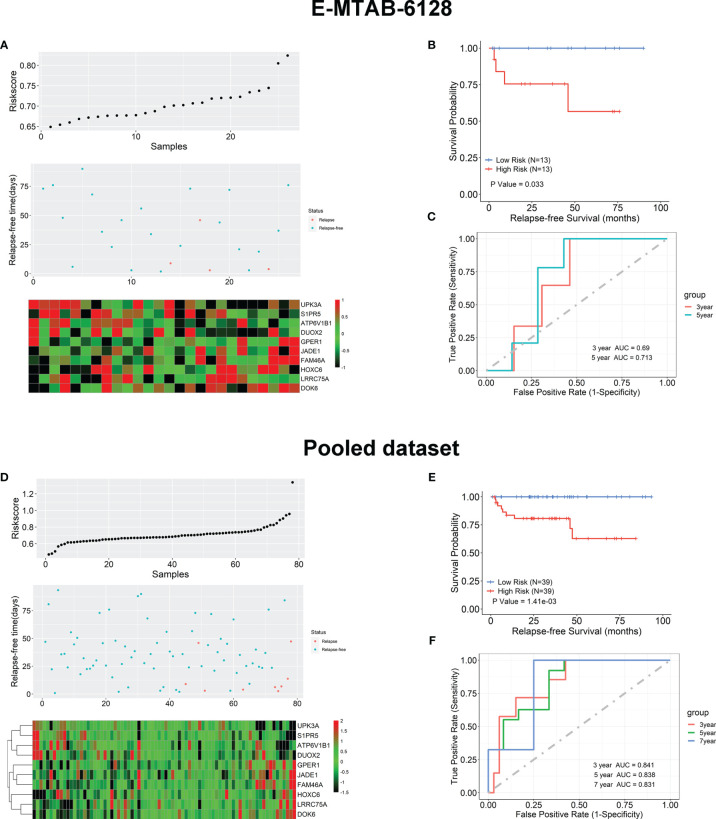
External validation of the 10-gene prognostic model using independent test set (E-MTAB-6128) and the pooled dataset (TCGA and E-MTAB-6128). **(A)** The risk score, survival status, and expression levels of the 10 genes based on the TAN samples in E-MTAB-6128. **(B)** Kaplan-Meier survival analysis in terms of BCR-free survival for the high- and low-risk groups in the TAN samples of E-MTAB-6128 database. **(C)** The ROC analysis for the prediction of the 3- and 5-year BCR-free survival based on the risk scores calculated by the 10-gene model. **(D)** The risk score, survival status, and expression levels of the 10 genes based on the TAN samples in the pooled dataset. **(E)** Kaplan-Meier survival analysis in terms of BCR-free survival for the high- and low-risk groups in the TAN samples in the pooled dataset. **(F)** The ROC analysis for the prediction of the 3-, 5-, and 7-year BCR-free survival based on the risk scores calculated by the 10-gene model.

We further verified the 10-gene TAN-tissue-based prognostic model using 26 TAN tissue samples from an independent test cohort (E-MTAB-6128). The exact formula (**Equation 1**) has been used to calculate the risk scores for each of these independent 26 patients. The relations between the expression of these 10 genes, the risk scores, and recurrence-free survival status are shown in **Figure 5A**. Similarly, these 26 patients were dichotomized into high- and low-risk groups based on the calculated median RS value, and the Kaplan-Meier analysis shown in **Figure 5B** indicated that these two groups had significantly different BCR outcomes (*p* = 0.033). The ROC analysis in **Figure 5C** showed that the prognostic accuracies for 3- and 5-year BCR-free survival were 0.68 and 0.713, respectively, for the 26 patients in E-TAB-6128 dataset. We then combined 52 patients in TCGA and 26 patients in E-TAB-6128 to form a large cohort and carried out the same test on this pooled data using the 10-gene prognostic model. The results are shown in **Figures 5D–F**, which also demonstrated the prediction value of the prognostic model in kthe TAN samples successfully.

### Bioinformatics Analysis of the 10 Prognostic Genes in Tumor Microenvironment

**Figure 6:**
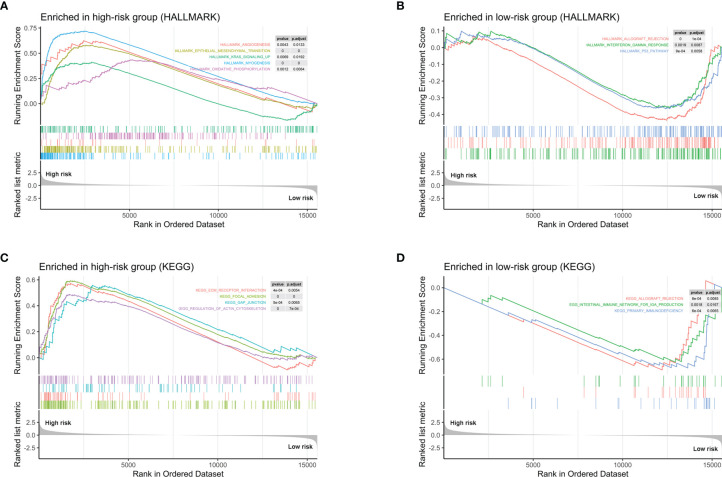
GSEA analysis between high- and low-risk patients determined by the 10-gene prognostic model, in the 52 TCGA-PRAD patients. **(A)** Hallmark gene sets strongly associated with the 10 prognostic gene in high-risk group. **(B)** Hallmark gene sets strongly associated with the 10 prognostic gene in low-risk group. **(C)** KEGG pathways that the 10 genes enriched in high-risk group. **(D)** KEGG pathways that the 10 genes enriched in low-risk group.

In order to explore the potential biological mechanisms involving these 10 prognostic genes, we conducted a GSEA analysis between high- and low-risk patients, determined by the 10-gene prognostic model, in the 52 TCGA-PRAD patients. Significant gene sets are shown in **Table 3** and the immune-related gene sets were visualized as an Enrichment Map (**Figure 6**). The results showed that these prognostic genes were strongly associated with the biological categories related to the carcinogenic pathway in the high-risk group, e.g., myogenesis, epithelial mesenchymal transition, angiogenesis, KRAS signaling, and oxidative phosphorylation, whereas, P53 pathways and some immune-related pathways, e.g., allograft rejection and interferon gamma response, were enriched in low-risk group (adjusted *p*-values <0.01 and normalized enrichment score (NES) >1 or NES <−1, **Figures 6A, B**). Similar result was obtained in the GSEA analysis between the high- and low-risk groups using the KEGG gene set. Carcinogenic pathways, e.g., ECM receptor interaction, focal adhesion, gap junction, and regulation of actin cytoskeleton, were enriched in high-risk group, and immune-related pathways, e.g., allograft rejection, intestinal immune network for IGA production, and primary immunodeficiency, were enriched in the low-risk group (adjusted *p*-values <0.01 and NES >1 or NES < −1, **Figures 6C, D**).

**Table 3:** GSEA for high- and low-risk groups based on 10-gene signature.

ID	Enrichment score	NES	*p*-Value	*p*-Adjust
Hallmark				
HALLMARK_MYOGENESIS	0.720	2.485	0.000	0.000
HALLMARK_EPITHELIAL_MESENCHYMAL_TRANSITION	0.580	2.034	0.000	0.000
HALLMARK_ALLOGRAFT_REJECTION	−0.435	−1.754	0.000	0.000
HALLMARK_TNFA_SIGNALING_*VIA*_NFKB	−0.406	−1.654	0.000	0.001
HALLMARK_UV_RESPONSE_DN	0.492	1.669	0.000	0.001
HALLMARK_INTERFERON_ALPHA_RESPONSE	−0.479	−1.797	0.000	0.002
HALLMARK_APICAL_JUNCTION	0.439	1.534	0.001	0.006
HALLMARK_P53_PATHWAY	−0.366	−1.487	0.001	0.006
HALLMARK_OXIDATIVE_PHOSPHORYLATION	0.435	1.522	0.001	0.006
HALLMARK_E2F_TARGETS	−0.365	−1.483	0.001	0.007
HALLMARK_INTERFERON_GAMMA_RESPONSE	−0.359	−1.469	0.002	0.009
HALLMARK_MYC_TARGETS_V2	−0.491	−1.689	0.002	0.010
HALLMARK_ESTROGEN_RESPONSE_EARLY	−0.347	−1.418	0.003	0.010
HALLMARK_INFLAMMATORY_RESPONSE	−0.366	−1.478	0.003	0.010
HALLMARK_ANDROGEN_RESPONSE	−0.416	−1.565	0.004	0.012
HALLMARK_ANGIOGENESIS	0.625	1.684	0.004	0.013
HALLMARK_PANCREAS_BETA_CELLS	0.663	1.649	0.007	0.019
HALLMARK_KRAS_SIGNALING_UP	0.412	1.435	0.007	0.019
KEGG				
KEGG_FOCAL_ADHESION	0.593	2.095	0.000	0.000
KEGG_RIBOSOME	−0.632	−2.340	0.000	0.000
KEGG_HYPERTROPHIC_CARDIOMYOPATHY_HCM	0.708	2.221	0.000	0.000
KEGG_DILATED_CARDIOMYOPATHY	0.686	2.172	0.000	0.000
KEGG_CALCIUM_SIGNALING_PATHWAY	0.571	1.953	0.000	0.000
KEGG_CARDIAC_MUSCLE_CONTRACTION	0.688	2.094	0.000	0.000
KEGG_REGULATION_OF_ACTIN_CYTOSKELETON	0.488	1.722	0.000	0.001
KEGG_ARRHYTHMOGENIC_RIGHT_VENTRICULAR_CARDIOMYOPATHY_ARVC	0.621	1.928	0.000	0.001
KEGG_VASCULAR_SMOOTH_MUSCLE_CONTRACTION	0.585	1.913	0.000	0.001
KEGG_CYTOKINE_CYTOKINE_RECEPTOR_INTERACTION	−0.411	−1.621	0.000	0.005
KEGG_GRAFT_*VERSUS*_HOST_DISEASE	−0.692	−1.960	0.000	0.005
KEGG_ECM_RECEPTOR_INTERACTION	0.573	1.803	0.000	0.005
KEGG_TYPE_I_DIABETES_MELLITUS	−0.683	−1.960	0.000	0.006
KEGG_GAP_JUNCTION	0.558	1.759	0.001	0.007
KEGG_PRIMARY_IMMUNODEFICIENCY	−0.674	−1.934	0.001	0.007
KEGG_ALLOGRAFT_REJECTION	−0.691	−1.912	0.001	0.009
KEGG_ADIPOCYTOKINE_SIGNALING_PATHWAY	0.561	1.711	0.001	0.015
KEGG_CYTOSOLIC_DNA_SENSING_PATHWAY	−0.582	−1.841	0.002	0.015
KEGG_INTESTINAL_IMMUNE_NETWORK_FOR_IGA_PRODUCTION	−0.625	−1.851	0.002	0.017
KEGG_NITROGEN_METABOLISM	0.744	1.798	0.002	0.020
KEGG_LONG_TERM_DEPRESSION	0.570	1.694	0.003	0.026
KEGG_PARKINSONS_DISEASE	0.474	1.586	0.003	0.026
KEGG_TYROSINE_METABOLISM	0.650	1.765	0.003	0.026
KEGG_NEUROACTIVE_LIGAND_RECEPTOR_INTERACTION	0.464	1.554	0.005	0.037
KEGG_GLYCEROLIPID_METABOLISM	0.586	1.692	0.006	0.044
KEGG_ASTHMA	−0.673	−1.700	0.006	0.044

## Discussion

There are not only morphological distinctions between tumors and their surrounding nontumor tissues but also many other forms of differences, including pH, allied gene imbalance and telomere length, stromal behavior, and transcriptional and epigenetic alteration. The formation of a tumor is typically associated with the alteration starting from the non-tumor components. The “field cancerization” theory on tumor microenvironment described a cumulative process of carcinogenesis in which genetic alterations are acquired stepwise, leading TAN tissues to an intermediate and preneoplastic state with morphologically normal but molecularly altered cells (20). Indeed, many studies have identified the differences in genomic transcription between healthy tissues and TAN tissues (10, 11). Since these transcriptional alterations in TAN tissues are caused by the nearby tumor tissue, our hypothesis is the profiles of such changes may reflect the aggressiveness of the tumor and, thus, may be leveraged as a signature for predicting cancer outcomes. Roman-Perez et al. described a multigene (>3,700) signature, derived from 72 extratumoral tissue with breast cancer, was capable of distinguishing active or inactive transcriptome phenotype, suggested that the phenotype of the extratumoral microenvironment may have value as an independent predictor of ER-positive/hormone-treated patient outcome (21). Wu et al. built a 73-gene signature of the tumor-adjacent parenchymal image feature which can stratify breast cancer patients into low- *versus* high-risk groups in terms of recurrence-free survival and overall survival (22). Besides, an advantage of using TAN samples as test material for disease prognosis is that TAN tissues have a simpler and more homogenous genetic background when compared with the tumor tissues. Therefore, if prognostic tests based on TAN samples cannot completely replace the tumor-based tests, they can at least serve as an efficient companion test to improve disease prognosis.

Many tumor-tissue based prognostic signatures have been constructed for prostate cancer. For example, Wang et al. used three Gleason score-associated genes to construct an outcome prediction model for prostate cancer (23). Hu et al. developed a prognostic and predictive model for overall survival and disease-free survival based on the five autophagy-related genes in prostate cancer (24). A clinical prediction model was built using three genes that are associated with prostate cancer BCR (25). One of our previous works based on the analysis of tumor tissues developed a 160-gene signature for predicting prostate cancer BCR (26). Moreover, Li et al. comprehensively evaluated the performances of machine learning models and 30 published prognostic signatures using PCa population cohorts of large sizes (27). In this study, we identified the DEGs between BCR patients and BCR-free patients using TCGA-PRAD cohort, and further screen these DEGs to establish a 10-gene prognostic model specifically for testing the TAN samples. The model can calculate an RS for each patient using the expression profiles of these 10 genes in TAN tissue, and the results showed that high-risk and low-risk group defined by the RSs had significantly different BCR-free survival. This model was further validated successfully using an independent dataset. The comparison between the prognostic genes in TAN tissues (in this study) and those in tumor tissues (26) showed that there was no overlap, suggesting potentially different mechanisms relevant to disease progression in these two tissue types. Several of the 10 genes in our TAN-tissue-based model have been reported to play critical roles in various types of cancer. For example, JADE1 plays a key role in HBO1 complex to regulate DNA replication initiation and, on the other hand, serves as a tumor suppressor by inhibiting proliferation and promoting apoptosis (28). In addition, JADE1 was stabilized by direct interaction with pVHL and directly linked to Wnt tumorigenesis pathway in renal cancer (29). UPK3A, which is specific to the urothelium, is involved in the process of epithelial cell differentiation and cell morphogenesis (30) and has been reported as a reliable marker for bladder cancer detection (31). It was reported that FAM46A protein was involved in cellular proliferation and associated with nonsmall cell lung cancer (32). Nishie et al. revealed the relationship between the expression of ATP6V1B1 and the intracellular environment of cancer cells, suggesting the downregulation of ATP6V1B1 affected the resistance to antibody-dependent cellular cytotoxicity (33). DUOX2, which promoted 5-fluorouracil-induced epithelial-mesenchymal transition by producing reactive oxygen species, appeared to play a significant role in colon cancer chemoresistance and the aggressiveness of this cancer (34). GPER1 was reported to be involved in the regulation of cellular growth, proliferation, and tumor development (35). Moreover, immunohistochemical studies have shown a positive association between the expression of GPER1 and the progression of female reproductive cancer (36).

Communication between tumor and surrounding histologically normal tissue, i.e., tumor microenvironment, is a two-way process, in which composite and complex mechanisms are involved. Identification of the commons and differences in biological pathways between these tissue types will advance our knowledge of cancer biology. The enrichment analysis showed that both the 160 prognostic genes in tumor tissues (26) and the 10 genes in the TAN-tissue-based model were significantly enriched by the p53 signaling pathway, indicating a potential crosstalk tunnel between these two types of tissues. Interestingly, “focal adhesion,” “gap junction,” and “adipocytokine signaling pathway” were found to be only activated in TAN tissues from BCR high-risk patients. The completion of tumor progression or tumor migration relies on intercellular communications *via* direct cell-to-cell contact or through paracrine/endocrine signaling, in which cytokines, chemokines, and growth factors represent the most common exchange molecules for signal transition, cell adhesion, and gap junctions (9). The results of the study indicated that the prognostic genes included in the 10-gene TAN-tissue-based model likely participate in prostate cancer progression in tumor microenvironment and, thus, they are useful biomarkers for the prediction of clinical outcomes of prostate cancer patients.

One limitation of the study is lacking a validation with a sufficiently large number of fresh samples which usually requires enormous effort in clinical practice, relevant management, such as specimen storage and follow-up with patient, and rigorous gene expression assay and data analysis. We are in the process of developing such a tissue bank and a database; however, they will not be available for a validation study in a few years. TAN tissues have not been brought to the research focus for decades, thus, only two datasets, i.e., TCGA-PRAD (RNAseq) and E-MTAB-6128 (Affymetrix Human Gene 2.0 ST Array), have been identified from public database to test our hypothesis aforementioned. Owing to the small number of TAN samples available for the study, we had limited statistical power to identify the prognostic genes with moderate or minor effects in tumor microenvironment. However, the results and findings of the study are very promising in spite of limited TAN samples, which warrants further exploration and clinical validation. Once more TAN tissues become available in the future, the advanced statistical methods, including BLUP-HAT, can be used to boost outcome predictability by including a large number of genes (from genes with major effects to genes with minor effect) in the regression model.

## Conclusion

This study developed a new 10-gene prognostic model for predicting biochemical recurrence-free survival leveraging gene expression profiles in tumor microenvironment. This innovative model has been rigorously validated using data from an independent cohort. Additional novelties of the study include: (1) Novel prognostic genes for prostate cancer have been identified in tumor microenvironment. (2) The potential roles of these prognostic genes in tumor microenvironment have been uncovered. (3) A common p53 signaling pathway that involves in prostate tumor progression has been detected between tumors and their microenvironment, indicating a potential crosstalk tunnel between these two tissue types.

## Data Availability Statement

Publicly available datasets were analyzed in this study. This data can be found here: the Cancer Genome Atlas (https://portal.gdc.cancer.gov/), the Gene Expression Omnibus (https://www.ncbi.nlm.nih.gov/geo/) and The ArrayExpress (https://www.ebi.ac.uk/arrayexpress/).

## Author Contributions

WZ and ZJ supervised the whole project and study and participated in study design and coordination. YF and RZ analyzed data and wrote and revised the paper. ZH, YL, QC, XX, YH, and JY revised the figures and verified the results. All authors contributed to the article and approved the submitted version.

## Funding

This work was supported by grants from the National Natural Science Foundation of China (82072813) and Guangzhou Municipal Science and Technology Project (201803040001) to WZ. The work was also supported by the UC Cancer Research Coordinating Committee Award, UC Riverside Hellman Fellowship, and UC Riverside start-up funding to ZJ; Natural Science Foundation of Guangdong Province (2020A1515010473) to ZH; Natural Science Foundation of Guangdong Province (2018A030313668) to YL; Zhejiang Provincial Medical and Health Science and Technology Project (2016KYB265), Ningbo Science and Technology Plan Projects (2016A610141), and Natural Science Foundation of Zhejiang Province (LY21H160015) to XX; and the China Postdoctoral Science Foundation 2020M682666 to JY.

## Conflict of Interest

The authors declare that the research was conducted in the absence of any commercial or financial relationships that could be construed as a potential conflict of interest.

## Publisher’s Note

All claims expressed in this article are solely those of the authors and do not necessarily represent those of their affiliated organizations, or those of the publisher, the editors and the reviewers. Any product that may be evaluated in this article, or claim that may be made by its manufacturer, is not guaranteed or endorsed by the publisher.

## Supplementary Material

Supplementary Figure 1 Expression profiles of 10 prognostic genes in TAN tissues (green dots) vs. prostate cancer tissues (orange dots) based on TCGAPRAD dataset.
