# Supplementary figures and images for: Prediction of Biochemical Recurrence-Free Survival of Prostate Cancer Patients Leveraging Multiple Gene Expression Profiles in Tumor Microenvironment
Source: Front Oncol. 2021 Sep 23;11:632571. doi: 10.3389/fonc.2021.632571 (PMC8495167; doi:10.3389/fonc.2021.632571)

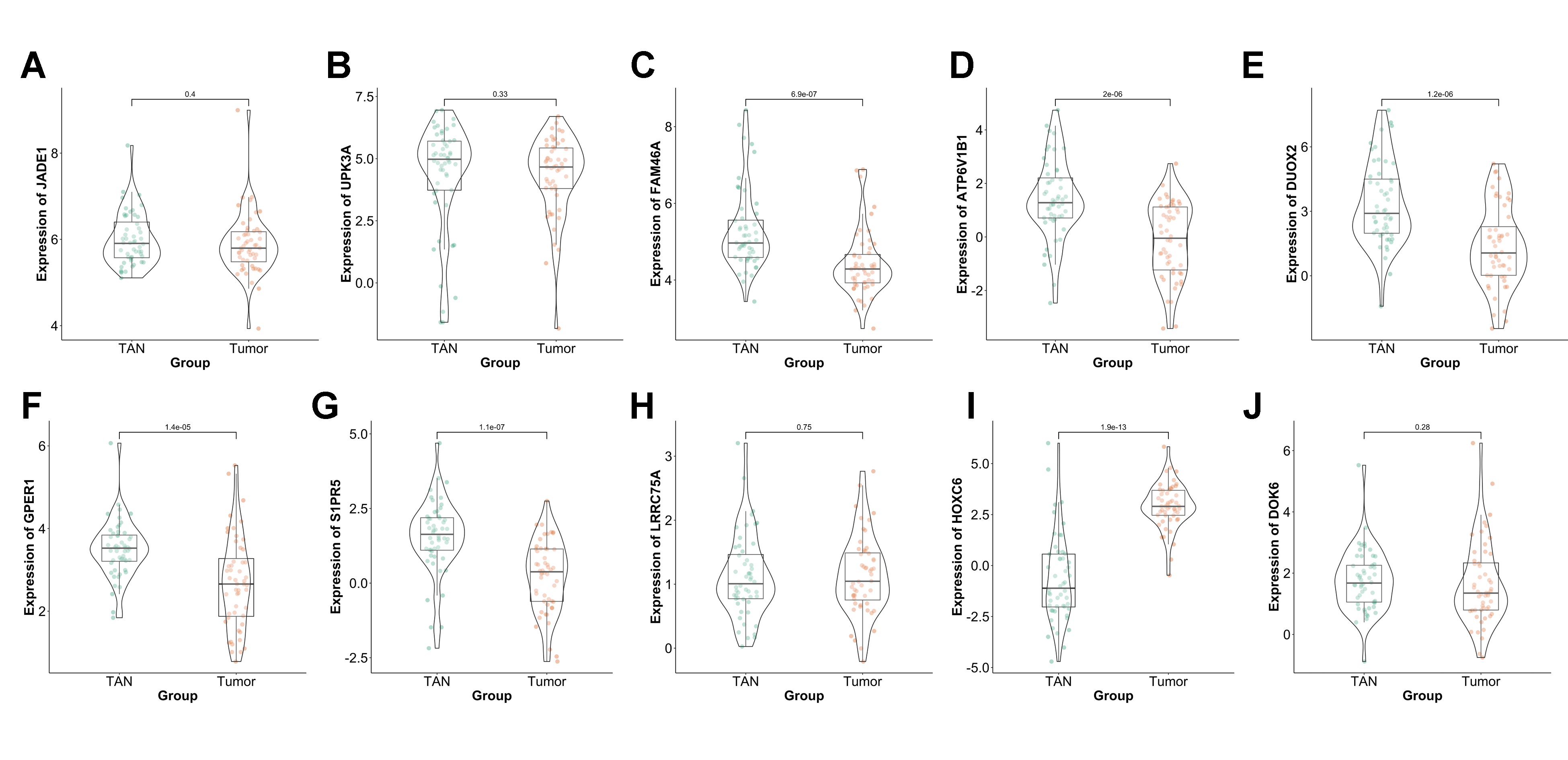

Supplement: Supplementary Figure 1 — Expression profiles of 10 prognostic genes in TAN tissues (green dots) vs. prostate cancer tissues (orange dots) based on TCGAPRAD dataset. [file Image_1.tif]
